# Sugar beet long-read reference assembly of genotype KWS2320

**DOI:** 10.1093/nargab/lqaf142

**Published:** 2025-11-13

**Authors:** Juliane C Dohm, Thomas Holzweber, Raphaela A Pensch, Heinz Himmelbauer

**Affiliations:** Institute of Computational Biology, Department of Biotechnology and Food Science, BOKU University, Muthgasse 18, Vienna 1190, Austria; Institute of Computational Biology, Department of Biotechnology and Food Science, BOKU University, Muthgasse 18, Vienna 1190, Austria; Institute of Computational Biology, Department of Biotechnology and Food Science, BOKU University, Muthgasse 18, Vienna 1190, Austria; Institute of Computational Biology, Department of Biotechnology and Food Science, BOKU University, Muthgasse 18, Vienna 1190, Austria

## Abstract

Sugar beet (*Beta vulgaris* ssp. *vulgaris*) is an important crop plant serving as a major source of sugar, particularly in Europe. Sugar beet research using the genotype KWS2320 has a long-standing history, and many datasets and studies exist that use this genotype as a reference. Here, we present a high-quality genome sequence of sugar beet genotype KWS2320 based on long-read sequencing data as well as an evidence-based gene set employing billions of mRNA (messenger RNA)-seq reads as transcript evidence. The assembly, referred to as RefBeet-3.0, was built using Pacific Biosciences data and was integrated with Bionano optical maps, Oxford Nanopore data, and various additional genomic resources. RefBeet-3.0 comprises 648 Mb in nine pseudochromosomes and further sequences with a total N50 size of 61.5 Mb. The gene set BeetSet-3 consists of 28 271 genes of which 25 824 could be functionally annotated based on sequence homology to orthologous groups. The assembly is highly complete and has a high sequence accuracy in absolute terms and in comparison to existing sugar beet assemblies. RefBeet-3.0 and BeetSet-3 will serve as comprehensive resources for future studies on sugar beet and other plants, as well as for breeding activities.

## Introduction

Beets of the genus *Beta* are plants affiliated with the family of Amaranthaceae, order Caryophyllales. The species of central importance within this genus is *Beta vulgaris* (*B. v*.) comprising all forms of cultivated beets in one subspecies *B. v. vulgaris*, i.e. sugar beet, fodder beet, leaf beet (or chard), and table beet (or red beet), as well as the wild subspecies *B. v. maritima* (sea beet) and *B. v. adanensis*. Together with two further species of wild beets, namely *B. macrocarpa* and *B. patula*, these taxa are considered as the primary gene pool in the genus *Beta*, encompassing the species and subspecies that can be crossbred with ease [1, 2]. All these beets are generally diploid with *n* = 9 chromosomes and have a haploid genome size of around 600–900 Mb [3, 4].

Beet crops universally are derived from sea beet, a mostly allogamous species widely distributed in the Mediterranean area, Macaronesia, and along the European Atlantic coast [5]. It was previously shown that Atlantic and Mediterranean specimens of sea beet are genetically distinct and can be separated by allele frequency statistics and by phylogenetic analysis [6–8]. Furthermore, cultivated beets as exemplified by sugar beet are most closely related to sea beets from the Eastern Mediterranean region, and in particular so from Greece, suggesting that the initial domestication of beets took place in this area [8].

The genome of sugar beet has been of long-standing interest to researchers and breeders. In an early strategic decision that was taken >20 years ago, efforts concentrated on the double-haploid (DH) sugar beet genotype KWS2320. Many molecular resources were generated using KWS2320, including transcript sequences from normalized complementary DNA (cDNA) libraries [9], genomic libraries in the format of bacterial artificial chromosome (BAC) clones [10] and fosmids [11], a first assessment of intraspecific diversity in beets [12], characterization of the repeat landscape of beets [13–19], genetic crosses for generating genetic maps of the sugar beet genome involving KWS2320 as one of the parents [20–22], and a physical BAC map of the beet genome [21]. Finally, the first high-quality beet reference genome assembly RefBeet-1.2 was based on the KWS2320 genotype [23]. The genome was assembled using a combination of whole-genome medium-long reads from the Roche 454 sequencing platform, Illumina mate-pairs (with up to 10 kb span size), Illumina PCR-free sequencing data, and end-sequences from KWS2320 fosmid clones (spanning ∼35 kb) and BAC clones (spanning ∼100 kb) generated with Sanger sequencing technology. The assembly was integrated with physical and genetic maps of the sugar beet genome including new data from a genotyping-by-sequencing approach [23]. At the time of its release, RefBeet-1.2 encompassed 567 Mb of which 85% were chromosomally anchored, including scaffolds that had chromosomal assignment, but lacked discrete position on the chromosome. The annotation of RefBeet-1.2 included the identification of repetitive sequences corresponding to 63% of the assembly length. The latest gene annotation of the RefBeet-1.2 assembly was performed using the Augustus software [24], employing gene prediction parameters optimized for Caryophyllales and based on training data obtained from Pacific Biosciences (PacBio) full-length transcript sequencing using the KWS2320 genotype. Gene prediction was supported by the inclusion of about four billion of Illumina mRNA (messenger RNA)-seq reads generated from different beet tissues and developmental stages (leaf, root, seed, seedlings, inflorescence) as well as from stressed plants. The final gene catalogue for RefBeet-1.2 encompassed 26 923 genes of which 65% were supported by 100% evidence, that is, indicating that all important gene features (start, stop, splice sites) were supported by cDNA sequencing data in these gene models, and was referred to as BeetSet-2 [25]. Gene names included chromosome identifiers, sequential numbers relating to the order of genes on the chromosome, and additionally a unique four-letter code providing stability during updates. Such stable identifiers greatly enhance the usefulness of a gene set, because previous and updated genome annotations can easily be linked, especially important in cases where beet gene names were included in the scientific literature.

Several other beet genome assemblies have been published. This includes assemblies from the wild beets *B. v. maritima* WB42, *B. patula* accession BETA 548, chard ‘White Silver’, and table beet W357B [26–28]. Assemblies for sugar beet are available for the DH lines KDHBv, UMSBv, YMoBv, and YTiBv, all of them assembled from Illumina paired-end and mate-pair data [23]. The genome of the inbred sugar beet genotype EL10 was assembled by leveraging a combination of PacBio sequencing, optical mapping, and Hi-C chromosome conformation capture [4]. Other sugar beet assemblies were prepared from long-read genomic sequencing data using Oxford Nanopore’s GridION sequencer. In particular, assemblies were generated for the lines U2Bv and KWS2320, both of which were integrated with a beet genetic map to provide long-range continuity [22, 29]. Nevertheless, based on the citation record, RefBeet remains the most widely used genomic resource of the beet genome. The Ensembl Plants database [30] also provides access to RefBeet.

In this work, we describe a new sugar beet assembly RefBeet-3.0 for the reference genotype KWS2320, based on PacBio long reads in combination with an optical map, and integrating resources that were used in the construction of former RefBeet versions. Additionally, recently published Oxford Nanopore data of KWS2320 [29] were included to further improve the contiguity of the assembly. We performed repeat annotation and established an evidence-based gene set BeetSet-3, whereby stable gene identifiers from BeetSet-2 were retained. The assembly and annotations of coding and non-coding genes are available at https://bvseq.boku.ac.at.

## Materials and methods

### Plant material, sequencing, and optical mapping

High-molecular weight DNA for PacBio sequencing was obtained from fresh sugar beet leaves of genotype KWS2320. The leaves were ground into powder under liquid nitrogen, followed by extraction of the genomic DNA using a Nucleospin XL kit (Macherey–Nagel, Düren, Germany).

At the Max Planck-Genome Centre in Cologne, Germany, PacBio sequencing was performed on the RSII sequencing instrument using P6-C4 sequencing chemistry (PacBio, Menlo Park, CA, USA). Long-read sequencing was performed on large SMRTbell gDNA libraries with inserts exceeding 15 kb after size selection. A total of 65 SMRT cells were sequenced, and a dataset of 4 670 776 polymerase reads was obtained.

Generation of an optical map on the Irys platform (Bionano Genomics Inc., San Diego, CA, USA) was contracted as a service from Bionano Genomics comprising DNA isolation and labelling, DNA quality control, data collection, *de novo* optical map assembly, and data review. DNA was isolated from 2.5 g of fresh leaves, followed by dual-motif nicking with Nt.BspQI and Nb.BbvCI, and single-colour labelling. Data collection was performed on 2.5 Irys chips, resulting in 88 GB of data encompassing molecules exceeding 150 kb with a single-molecule N50 length of 229 kb. Raw data were converted to single-molecule maps using the RefAligner software (Bionano Genomics), and an assembly of 591 Mb was obtained, with an N50 size of 1.12 Mb; the longest optical map contig had a length of 4.9 Mb.

### Genome assembly

Starting from 5 504 676 PacBio sequencing reads (subreads), we assembled the data using canu v2.0 [31] and parameters corOutCoverage=100 correctedErrorRate=0.03 into an initial assembly comprising 3634 sequences (longest sequence: 11.5 Mb) with a total length of 650.9 Mb and an N50 size of 2.6 Mb. Previously generated end sequences of BACs and fosmids using the Sanger sequencing technology comprised 20 682 pairs of BAC ends (mean span size ∼100 kb) and 90 433 pairs of fosmid ends (mean span size ∼35 kb) [23]. These sequence pairs were mapped to the initial PacBio assembly using BLASR v5.1 [32] with parameters -m 5 --bestn 1 --nproc 18, and the information of pairs that matched at contig ends and established links between contigs was extracted. Sequence-based genetic markers as used for RefBeet-1.2 [23] were located in the assembly with standalone BLAT v35 [33] using default parameters, and the result was filtered for the best matching location. The contigs were ordered according to genetic markers, and for each pair of neighbouring contigs it was checked if linking BAC and/or fosmid ends existed based on which the distance of the contigs was estimated. In many cases the result was a negative distance pointing to overlapping contig ends, and nucmer from mummer suite v3.1 [34] was used to align such ends to each other (focusing on the last 30 kb of two overlapping contigs) and to determine the length of the overlap. The nucmer parameters were --maxmatch -b 1000 with delta-filter -i 95 -l 100 -g and show-coords -ldT. Contigs were merged according to their overlap and in agreement with the estimated distance based on BAC and fosmid ends. If no overlap could be found, the contigs were scaffolded by introducing an N-gap of a length according to the estimated distance or of length 10 if the estimated distance was negative. During this process a number of misassemblies were detected, and such contigs were cut at a likely position of the misassembly determined manually by evaluating the positions of mapped single reads and paired sequences. The assignment of scaffolds to chromosomes was indicated by the genetic markers, and further confirmation of chromosomal assignment was obtained from mapping short-read data from 15 progeny of an F2 generation to sequences of a minimum size of 50 kb following the steps previously described but without using the parent line ‘P2’ [23]. A few more sequences could be chromosomally assigned based on the variant patterns of these data.

For the integration of this intermediate assembly with optical mapping data, we first performed an *in silico* digest of the assembly using knickers v1.5.5 (Bionano Genomics) with Nt.BspQI and Nb.BbvCI as nicking enzymes. The comparison with optical maps was run using the RefAligner program (Bionano Genomics) with parameters -f -endoutlier 1e-3 -outlier 1e-4 -extend 1 -FN 0.05 -FP 0.5 -sf 0.2 -sd 0.1 -sr 0.02 -res 2.9 -resSD 0.75 -mres 1.2 -A 5 -biaswt 0 -M 1 -Mfast 0 -maxmem 128 -maxthreads 32 -deltaX 9 -deltaY 9 -xmapchim 14 -xmapUnique 14 -RepeatMask 4 0.01 -RepeatRec 0.7 0.6 1.4 -T 1e-10 -hashgen 5 3 2.4 1.5 0.05 5.0 1 1 2 -hash -hashdelta 10. Optical mapping contigs that were found to be composed of repetitive sequence (high coverage and dispersed matches) were ignored. The gap size between sequences was determined based on the resulting xmap file, and the corresponding number of Ns was inserted between connected sequences. If the optical map comparison indicated overlapping sequences, 100 Ns were inserted between them. Manual confirmation was done using the IrysView viewer v2.5.1.29842 (Bionano Genomics).

We checked whether contigs were redundant using minimap2 [35] and removed smaller contigs in case they were located inside larger contigs, which was mainly found at overlap regions (126 contigs, summing up to 2.3 Mb, were removed). Isogenic Illumina paired-end data [23] were used for polishing the assembly. The reads were mapped with bowtie2 [36] using the parameters -p 24 --no-unal -t --no-discordant -I 530 -X 660 --gbar 2 -S; the resulting sam file was sorted and indexed using samtools v1.3 [37], and six rounds of polishing were performed using pilon v1.22 [38] called with java -Xmx750G -jar pilon-1.22.jar and the parameters --fix bases --changes --tracks --threads 20 --flank 2.

The resulting intermediate assembly was compared to the sugar beet assembly of genotype EL10 [4], version EL10.2, using minimap2 and visualized using circlize v0.4.15 [39]. Differences were manually inspected and revised: 3 scaffolds were split into two parts, 15 scaffolds of previously unknown orientation were flipped, and 12 previously chromosomally assigned but unanchored scaffolds were located within chromosomes. Comparison with the assembly 2320BvONT [29], based on the same genotype as RefBeet, confirmed all splits and most of orientations and locations. Since the construction of 2320BvONT included the same genetic markers, some regions remained unanchored or unassigned there, too, and could only be located using the assembly of the EL10 genotype. We assembled PacBio data (this study) and Nanopore data (NCBI SRA: ERR3950579-ERR3950598; 4 095 356 reads in total) of KWS2320 together using canu v2.2 (parameters: genomeSize=750m corMhapFilterThreshold=0.0000000002 corMhapOptions=“--threshold 0.80 --num-hashes 512 --num-min-matches 3 --ordered-sketch-size 1000 --ordered-kmer-size 14 --min-olap-length 2000 --repeat-idf-scale 50″ mhapBlockSize=500 ovlMerDistinct=0.975), followed by polishing using pilon. The result of this PacBio–Nanopore assembly was mapped against the so far ordered and orientated sequences (referred to as RefBeet-1.95) using minimap2 with parameters -c and -x asm5. We manually inspected potential connections within RefBeet-1.95 based on bridging sequences of the PacBio–Nanopore assembly. Connections were established based on mapping positions (manually) and using the PacBio–Nanopore sequence for gap-filling with RagTag v2.1.0 [40] to receive a version of improved contiguity. During this process, contig ends were removed in RefBeet if they were in disagreement with continuous connecting sequences, and three scaffolds were cut and rearranged according to the PacBio–Nanopore assembly. We joined RefBeet sequences into pseudochromosomes with 1 kb long N-stretches between sequences to generate the final version RefBeet-3.0.

### Assembly quality assessment

BUSCO v5.8.3 with datasets eukaryota_odb12 and eudicots_odb12 [41] was used for completeness estimation (standard parameters and --miniprot). The sequence of the BAC ZR47B15, Genbank accession ID FJ752587, was mapped against the assemblies using makeblastdb and blastn [42]. Gaps and mismatches were extracted from the blast output (format -outfmt 6).

### Repeat annotation

Repeat masking of the RefBeet-3.0 assembly was performed with RepeatMasker v4.1.6 [43] using a *de novo* repeat catalogue generated by RepeatModeler v2.0.5 with default parameters and -LTRStruct [44], and the databases Dfam v3.8 [45] and Repbase v20181026 [46]. For gene prediction, unknown repeats as well as simple and low-complexity sequences remained unmasked.

### Gene prediction and functional annotation

As input for gene prediction, we used the soft-masked sequence of RefBeet-3.0 (no masking of unknown, simple, low complexity sequences) and transcription evidence from 3 988 584 663 mRNA-seq reads of *B. v. vulgaris* (NCBI SRA accession numbers: ERR11534264-ERR11534303, ERR11534320, ERR11534330-ERR11534345, SRR1508751, SRR1508753, SRR1508755, SRR1508756, SRR1508758, SRR868886, SRR868887, SRR869751-SRR869758; example link to the data: https://www.ncbi.nlm.nih.gov/sra/?term=ERR11534264) and from 5 310 477 protein sequences of *Viridiplantae* as included in OrthoDB v11 [47]. RNA mapping was performed with hisat2 v2.2.1 [48]. The software BRAKER3 [49] was run for evidence-based gene prediction in ETPmode with default parameters and --augustus_args=“--noInFrameStop=true --genemodel=complete” --busco_lineage=eudicots_odb10 --species=beta_vulgaris --useexisting, where beta_vulgaris refers to previously determined taxon-specific parameters [25]. With the default filtering step of BRAKER3, only transcripts with 100% intron evidence were retained. BUSCO analysis was applied on the unfiltered and filtered gene sets using the eudicots_odb10 lineage dataset. The result indicated that filtering led to the loss of 11 BUSCOs (0.47%). These sequences were subsequently reintegrated from the unfiltered into the filtered gene set. Completeness analysis on the final gene set used both the eukaryota_odb12 and eudicotyledons_odb12 lineage datasets. UTRs were annotated using stringTie [50]. For functional annotation of the resulting genes, we used eggNOG mapper v2.1.12 [51] and eggNOG DB v5.0.2 [52]. Liftoff v1.6.3 [53] was used to transfer BeetSet-2 gene annotations to the RefBeet-3.0 genome assembly (mapping of gene models to the assembly), and blastn v2.16.0 was used to compare BeetSet-2 coding sequences to BeetSet-3 genes. If a BeetSet-2 gene was overlapping with a BeetSet-3 gene (according to the Liftoff result) and showed similarity to the same gene (according to blastn), the stable identifier from BeetSet-2 was transferred to the corresponding BeetSet-3 gene model. AGAT v1.4.0 [54] was used for formatting the gff file and extraction of the coding sequence and the amino acid sequence. Final sorting of the gff file was done using Linux tools (sed, sort).

Non-coding RNAs were predicted using cmscan from INFERNAL v1.1.5 with Rfam covariance models (release 14.5) and settings --cut_ga --rfam --nohmmonly -clanin Rfam.clanin --oskip --fmt 2 --tblout [55, 56]. An additional tRNA (transfer RNA) analysis was performed using tRNAscan-SE v.2.0.12 [57] with domain search mode -E.

### Computing resources and data management

Data were analysed using a Linux cluster running CentOS 6, CentOS 7, and AlmaLinux 8 on nodes with up to 64 cores and at most 1 TB RAM. Additionally, analyses were performed on the Vienna Scientific Cluster (VSC-3 and VSC-5) using up to 128 cores and 1 TB RAM.

For general tasks to organize files and sequences, we used common Linux tools, bioawk [58], parallel [59], seqtk [60], SeqKit2 [61], JBrowse2 [62], Perl 5 [63], and Python 3 [64].

## Results

### Assembly construction

We sequenced the sugar beet genotype KWS2320 using the PacBio technology RSII at an estimated genome coverage of 60× and generated Bionano optical mapping data. The raw PacBio data were quality-filtered and assembled into an initial assembly of 651 Mb total size and a contig N50 of 2.6 Mb. For preparation of an optical map, dual motif labelling on the Bionano Genomics (BioNano) Irys system [65] resulted in the collection of molecules exceeding 150 kb equivalent to 117× genome coverage. An optical map was calculated encompassing 591 Mb and a BioNano contig N50 size of 1.12 Mb. Integration of the initial PacBio assembly with previously generated BAC-end sequences, fosmid-end sequences, genetic markers, genotyping-by-sequencing data [23], and with the BioNano optical map increased the contig and scaffold N50 to 10.6 Mb. The contiguity was further improved by including Oxford Nanopore data of the same genotype KWS2320 [29]. After polishing using Illumina data and removal of contamination, the assembly comprised 3140 contigs and scaffolds with an N50 size of 13.7 Mb with a longest sequence of 41.9 Mb (belonging to chromosome 6). Comparison to the pseudochromosomes of sugar beet assembly EL10.2 [4] confirmed the order and orientation of sequences in most cases and allowed the placement of some of the chromosomally assigned but unanchored sequences inside chromosomes. The adapted order was confirmed by integrating Nanopore data of KWS2320. The final assembly, referred to as RefBeet-3.0, consisted of nine pseudochromosomes and 3096 smaller sequences with a total assembly size of 647.6 Mb and an N50 size of 61.5 Mb. The largest sequence was pseudochromosome 6 (71.4 Mb) and the smallest was a 1105 bp long contig (Table 1). Apart from the pseudochromosomes, 19 further sequences were chromosomally assigned (in total 4.7 Mb), and mitochondrial and chloroplast sequences were identified. RefBeet-3.0 was submitted to the NCBI genomes database, accession number JBNBID000000000.

**Table 1.** Assembly metrics of RefBeet-3.0 and three previously published sugar beet assemblies. RefBeet-3.0 has the highest fraction of complete genes indicating its high quality

	RefBeet-3.0(this work)	2320BvONT[29]	EL10.2[4]	RefBeet-1.2[23]
Assembly size (Mb)	647.6	573.0	568.8	566.6
# Sequences	3105	79	18	40 508
# Sequences ≥ 100 kb	138	79	17	461
N50 size (Mb)	61.5	54.4	62.0	2.0
Longest sequence (Mb)	71.4	71.8	72.5	10.4
Shortest sequence (kb)	1.1	102.6	81.4	0.5
N50 size before pseudochromosomes (Mb)	13.7	9.5	-	-
Longest sequence before pseudochromosomes (Mb)	41.9	29.7	-	-
				
BUSCO completeness Eukaryota (129 genes) (%)				
Complete	99.2	98.4	96.9	96.9
Single-copy	89.9	89.1	86.8	86.8
Duplicated	9.3	9.3	10.1	10.1
Complete with internal stop	1.6	0.8	0.8	0.8
Fragmented	0.8	1.6	1.6	3.1
Missing	0.0	0.0	1.6	0.0
				
BUSCO completeness Eudicots (2805 genes) (%)				
Complete	97.2	97.1	95.9	97.1
Single-copy	95.7	95.5	94.2	95.7
Duplicated	1.5	1.6	1.7	1.5
Complete with internal stop	2.0	1.9	2.3	2.0
Fragmented	1.6	1.6	1.4	1.6
Missing	1.2	1.4	2.7	1.2

### Assembly quality and comparisons

We searched for conserved genes using Benchmarking Universal Single-Copy Orthologs (BUSCO) [66] to assess the completeness of the RefBeet-3.0 assembly. The detected 99.2% complete conserved genes of eukaryota and 97.2% complete conserved genes of eudicots indicated that RefBeet-3.0 was highly complete (Table 1). These values were slightly higher than in the assemblies 2320BvONT [29] and EL10.2 [4]. In the previous version RefBeet-1.2 with a scaffold N50 size of only 2 Mb, the complete conserved genes were found at fractions similar to the ones of the 2320BvONT and EL10.2 assemblies (Table 1) indicating that RefBeet-1.2 contained an almost complete gene space despite its rather small N50 size.

We assessed the sequence accuracy by aligning a BAC clone (ZR47B15, length 109 563 bp), isogenic and fully sequenced by Sanger technology [12], against the assemblies. In RefBeet-3.0, the BAC sequence was aligned in one piece in chromosome 1 showing zero mismatches and nine gaps. These gaps had a size of one base each and were within homopolymer stretches, i.e. five times one missing nucleotide in G-stretches, two times in C-stretches, and one missing T and one additional T, respectively, in two T-stretches. The alignment of ZR47B15 to the 2320BvONT assembly resulted in 15 mismatches and 23 gaps of various lengths of up to ten bases. In the previous version RefBeet-1.2, the alignment of ZR47B15 was split into three matches of lengths 32, 69, and 8 kb, respectively, with a total of 12 mismatches and 11 gaps in addition to the gaps between matches. In the EL10 genotype, we expected more differences and found nine separate matches exceeding 2 kb (longest match 35 kb) with a total of 784 mismatches and 243 gaps within the aligning sequence.

The end sequences of previously determined terminal BACs [67] could be located in the pseudochromosomes of RefBeet-3.0 resulting in matches at distances between 13 kb and 1.7 Mb from the beginning of the assembled chromosomes for ‘northern’ BACs and between 100 kb and 1.9 Mb distance from the end of chromosomes for ‘southern’ BACs. Further sequence-based genetic markers that were used to support the construction of RefBeet-3.0 were mapped back to the final assembly to determine their positions (Supplementary Table S1). In six chromosomes, the terminal BACs showed the furthest ‘northern’ matching positions and in three chromosomes the furthest ‘southern’ matching positions compared to all genetic markers. In the remaining cases, there was at least one of the genetic markers that matched closer to the end of the chromosome than these BACs.

**Figure 1.**
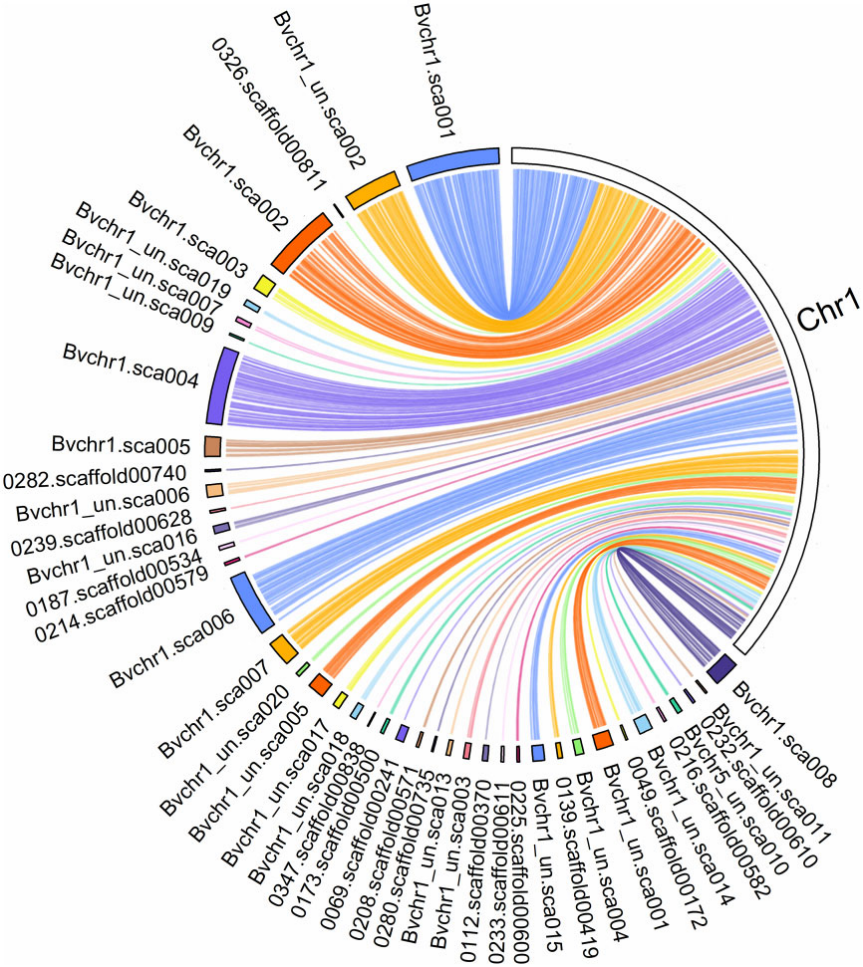
Comparison between RefBeet-1.2 (left side, coloured) and RefBeet-3.0 chromosome 1 (right side, white). Sequences with matches of a minimum length of 50 kb and a minimum mapping quality of 50 are shown. The order of chromosomally anchored sequences in RefBeet-1.2 was confirmed and the location of 17 scaffolds previously assigned to chromosome 1 but unanchored could be resolved. Additional scaffolds previously not assigned to chromosome 1 could be located.

Sequences of the previous assembly version RefBeet-1.2 have been in use over the past ten years in many different projects, resulting in 758 citations (google scholar, October 2025). Now, these sequences could be ordered and orientated along RefBeet-3.0. We mapped the two assemblies onto each other and provide a table of match positions and a graphical overview to link the sequences of the two versions to each other in order to provide continuity and to promote a smooth transition between the two assembly versions (Fig. 1, Supplementary Fig. S1, and Supplementary Table S2).

**Figure 2.**
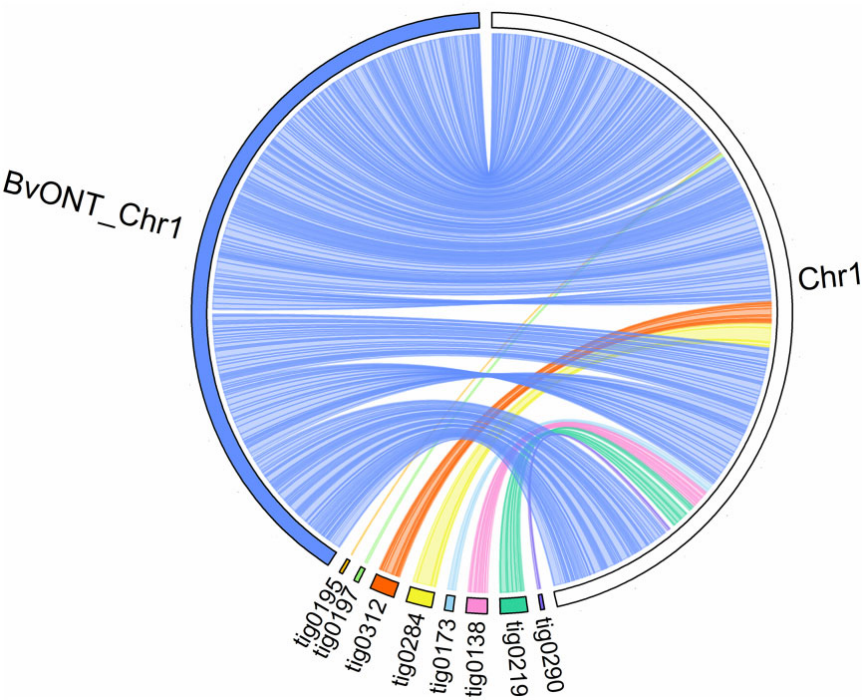
Comparison between 2320BvONT (left side, coloured) and RefBeet-3.0 chromosome 1 (right side, white).

**Figure 3.**
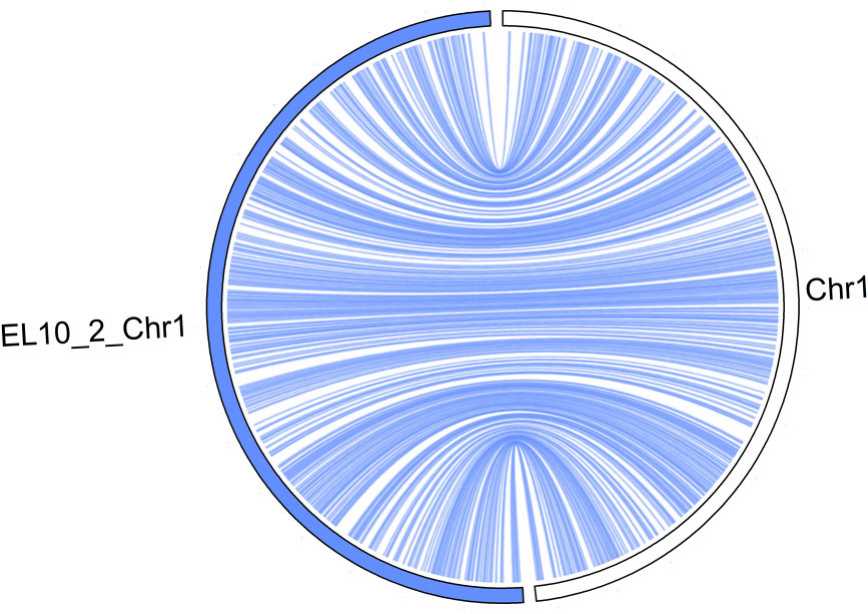
Comparison between EL10.2 (left side, coloured; inverted) and RefBeet-3.0 chromosome 1 (right side, white).

Comparisons of RefBeet-3.0 pseudochromosomes to the assembly 2320BvONT showed largely agreement (Fig. 2 and Supplementary Fig. S2). In cases where RefBeet-3.0 and 2320BvONT differed from each other, the order and orientation of RefBeet-3.0 was confirmed by genotype EL10 (apart from the entirely reversed orientation of chromosomes 1, 2, and 4 in the assembly EL10.2) and by connecting Nanopore sequencing reads (Fig. 3 and Supplementary Fig. S3). In cases where initial assembly versions of RefBeet differed from EL10, 2320BvONT confirmed EL10 so that we could safely adapt RefBeet accordingly (see ‘Materials and methods’ section). Larger differences between RefBeet-3.0 and 2320BvONT were detected in the assemblies of chromosomes 3 and 4, whereas, for example, chromosomes 6 and 7 were very similar (Supplementary Fig. S2).

Based on homology searches using comprehensive repeat databases and using a collection of inherently detected repeats of RefBeet-3.0, we found a repetitive fraction of 69.1% in the assembly. The largest group of repeats were retroelements, most prominently long terminal repeat elements (Supplementary Table S3).

### Gene prediction and functional annotation

We performed gene prediction in RefBeet-3.0 using transcription evidence from approximately four billion Illumina mRNA-seq reads from *B. v. vulgaris* and five million Viridiplantaeprotein sequences. Repeats were masked in the assembly before genes were predicted. The predictions resulted in a gene set of 28 271 genes (32 609 transcripts, all supported by 100% intron evidence), referred to as BeetSet-3. The average coding sequence length was 1106 bp and the average number of exons per gene was 4.4. Most genes (24 422) had a single transcript, 3463 had two transcripts, and the largest number of transcripts per gene was eight (three genes). The completeness of BeetSet-3 assessed based on BUSCO was 100% for conserved genes of eukaryota and 98.2% for conserved genes of eudicots (Table 2), which were the highest fractions compared to gene sets of the 2320ONT and EL10.2 assemblies as well as compared to BeetSet-2 [25] predicted based on the RefBeet-1.2 assembly.

**Table 2.** Assessment of the completeness of gene predictions in RefBeet-3.0 (referred to as BeetSet-3) and three previously published sugar beet assemblies. BeetSet-3 has the highest fraction of complete genes compared to the other gene sets

	BeetSet-3(this work)	Gene set of 2320BvONT [29]	Gene set of EL10.2 [4]	BeetSet-2 [25]
Number of genes	28 271	26 364	21 587	26 923
BUSCO completeness Eukaryota (129 genes) (%)				
Complete	100.0	95.3	97.7	97.7
Single-copy	81.4	69.8	84.5	82.2
Duplicated	18.6	25.6	13.2	15.5
Fragmented	0.0	3.9	0.0	2.3
Missing	0.0	0.8	2.3	0.0
BUSCO completeness Eudicots (2805 genes) (%)				
Complete	98.2	91.8	95.5	96.6
Single-copy	78.6	74.7	84.9	87.1
Duplicated	19.6	17.1	10.6	9.6
Fragmented	0.6	5.7	1.4	1.8
Missing	1.2	2.5	3.1	1.6

Stable identifiers (four-letter codes) of 19 246 genes in BeetSet-2 were transferred to the genes in BeetSet-3 after detailed comparisons (see ‘Materials and methods’ section) for convenient recognition of corresponding genes. Additionally, 9025 new stable identifiers were assigned to the remainder of predicted BeetSet-3 genes. The identifier of a single gene of special interest, the rhizomania resistance gene *Rz2* [68] annotated in BeetSet-1 [23] and missing in BeetSet-2, was manually assigned to the corresponding gene present in BeetSet-3 (‘jumg’, located on pseudochromosome 3). No further comparisons to BeetSet-1 were performed.

The gene set was functionally annotated using homology searches in a comprehensive database of orthologous groups (OGs) from various species and by transferring the functions of the orthologs to BeetSet-3 transcripts. In total, 30 002 of the predicted transcripts (25 824 genes) received functional information of which 26 290 transcript annotations were based on OGs from the taxonomic level of Streptophyta, 2059 based on OGs from eukaryota, and 759 based on Viridiplantae OGs as the three largest groups. Functional descriptions were assigned to 27 358 transcripts (23 561 genes) and gene ontology terms to 14 497 transcripts (12 278 genes). Assignments to functionally annotated COGs (clusters of orthologous genes) were obtained for 17 933 transcripts (15 391 genes) of BeetSet-3 (Supplementary Fig. S4).

Annotation of non-coding RNAs resulted in 6602 loci of which 3383 were in chromosomally assigned scaffolds. Specifically, we annotated 1092 tRNA loci, 3715 loci encoding ribosomal RNAs (nuclear and mitochondrial), 1203 loci encoding small nucleolar RNAs, 200 loci encoding microRNAs, 180 group II catalytic introns, and 152 spliceosomal RNAs as most abundant groups, whereby several non-coding RNAs were encoded by multiple genes. A more detailed tRNA analysis resulted in 1112 tRNA predictions of which 1005 were decoding the 20 standard amino acids, four were predicted as suppressor tRNAs, and 68 as pseudogenes.

## Discussion

We here present a new and greatly improved sugar beet genome assembly RefBeet-3.0 to replace previous versions of RefBeet. Persistent and high-quality annotation is a key feature of a genome assembly regarding its usefulness for the scientific community. For instance, despite massive advances in genetics and genomics, the currently most widely used assembly version for the human genome is GRCh38/hg38 released more than ten years ago in December 2013 [69], although a more complete human genome sequence released by the telomere-to-telomere consortium is available [70]. To ensure that the new RefBeet genome assembly will readily be adopted by researchers working on beets, we provide a comparison of RefBeet-3.0 and RefBeet-1.2 assemblies in tables and graphics. Furthermore, we have used persistent identifiers for those genes in the new gene set BeetSet-3 that had already been annotated in RefBeet-1.2 (gene set BeetSet-2). Gene names as published with the assemblies 2320BvONT and U2BvONT [29] were inspected to avoid double usage of identifiers. The process of comparing previous and new gene predictions may differ for the gene set established in 2320BvONT; however, we can guarantee consistency between BeetSet-2 and BeetSet-3 and uniqueness of identifiers compared to further genes predicted in 2320BvONT v1 and v2 and in U2BvONT. In total, 19 246 genes in BeetSet-3 share the identifier with their corresponding gene model in BeetSet-2, while 9025 genes were ‘new’ in BeetSet-3 (i.e. had no obvious counterpart in BeetSet-2 when comparing the two gene sets) and received a new persistent identifier. Previously predicted 7677 genes of BeetSet-2 were no longer included (e.g. in case gene fragments had been predicted separately in BeetSet-2 and were merged into a complete gene in BeetSet-3) and their identifiers were discontinued. The measure of providing consistent identifiers will promote the smooth transition to RefBeet-3.0.

RefBeet-3.0 contains nine pseudochromosomes, equivalent to the known haploid chromosome number in beets, encompassing most of the assembly (86%). Compared to previous assemblies of genotype KWS2320, RefBeet-3.0 has the largest N50 size before and after building pseudochromosomes. No size cutoff was applied so that smaller contigs were kept in RefBeet-3.0. We noticed that genes were predicted even in such small contigs. The smallest contig containing a predicted gene (or gene fragment) was 2773 bp in length, so we ensured a comprehensive gene set by keeping all assembled contigs.

We compared RefBeet-3.0 to the assembly EL10.2 generated from the genotype EL10, an inbred derivative of sugar beet accession C869 (PI 628755) [4]. We observed collinearity between the two assemblies over most regions of the beet genome, but also noted several small-scale discrepancies as exemplified in the matching region of the BAC sequence derived from genotype KWS2320. Such differences can be most plausibly interpreted as variation that exists in the beet gene pool.

To assess sequencing accuracy among assemblies of the genotype KWS2320, we compared the same BAC clone ZR47B15 (sequenced with Sanger technology) with RefBeet-3.0 (PacBio), 2320BvONT (Oxford Nanopore), and an internal assembly that used both PacBio and Nanopore data as input. While there was consistently high sequence quality in the assemblies, the smallest number of mismatches was recorded for RefBeet-3.0. All mismatches were located within homopolymer stretches. The question remains whether the Sanger sequence of the BAC or the PacBio-based sequence (after Illumina-based polishing) of RefBeet-3.0 is the correct version, considering that neither Illumina nor Sanger sequencing technologies are entirely without errors when it comes to homopolymer sequencing [71, 72].

The discovery and annotation of transcribed sequences in a newly sequenced genome is of primary concern, and decisions about how to perform gene annotation can profoundly alter the outcome. During annotation of the previous RefBeet version (resulting in BeetSet-1 and BeetSet-2) different strategies had been employed. Generation of BeetSet-2 involved model training in order to optimize the gene prediction for Caryophyllales, increasing the quality of gene prediction in comparison to the previous gene set. On the other hand, transposable elements (TEs) were filtered out more aggressively than for the generation of BeetSet-1. In this way, the TE-containing locus Bv3u_069081z4260_jumg that predisposes to rhizomania on beet chromosome 3 [68] was annotated with a gene model in BeetSet-1 but was not retained in BeetSet-2. In the newly established BeetSet-3, we employed soft-masking (changing repetitive sequence to lower-case rather than to N stretches), thus preserving potential coding boundaries, and the TE-containing locus was again annotated in BeetSet-3 (as Bv3_067 990_jumg on pseudochromosome 3, start codon at position 9559786).

## Conclusion

We here provide a high-quality sugar beet genome assembly RefBeet-3.0 from the DH reference genotype KWS2320 in nine pseudochromosomes. The assembly is based on PacBio long-read sequencing data that were combined with optical maps, further genetic and physical data as well as Illumina data from previous projects, and with recently published Nanopore sequencing data available for this beet genotype. Comparisons and stable gene identifiers were used to link RefBeet-3.0 and its gene prediction to a previous version of RefBeet, providing continuity. The high quality of the assembly combined with ease of access to sequences and annotations will make RefBeet-3.0 a widely used molecular resource for researchers working on sugar beet and its relatives.

## Supplementary Material

lqaf142_Supplemental_Files

## Data Availability

PacBio raw data are available at the NCBI SRA (BioProject PRJNA1233215, BioSample SAMN47263249). The RefBeet-3.0 assembly is available at NCBI genomes (accession number JBNBID000000000). The assembly as well as gene predictions (coding genes and non-coding RNAs) are available for download at https://bvseq.boku.ac.at.

## Notes

Present address: Department of Medical Biochemistry and Microbiology, Uppsala University, Dag Hammarskjölds väg 20, Uppsala 751 85, Sweden
