# Supplementary material for: Sugar beet long-read reference assembly of genotype KWS2320
Source: NAR Genom Bioinform. 2025 Nov 13;7(4):lqaf142. doi: 10.1093/nargab/lqaf142 (PMC12614215; doi:10.1093/nargab/lqaf142)
Supplement: lqaf142_Supplemental_Files [file lqaf142_supplemental_files.zip › Supplement_BvPacBio_revision_v5.pdf]

Sugar beet long-read reference assembly of genotype KWS2320

Juliane C. Dohm[1]*, Thomas Holzweber[1]*, Raphaela A. Pensch[1,2], Heinz Himmelbauer[1]

Supplementary Information

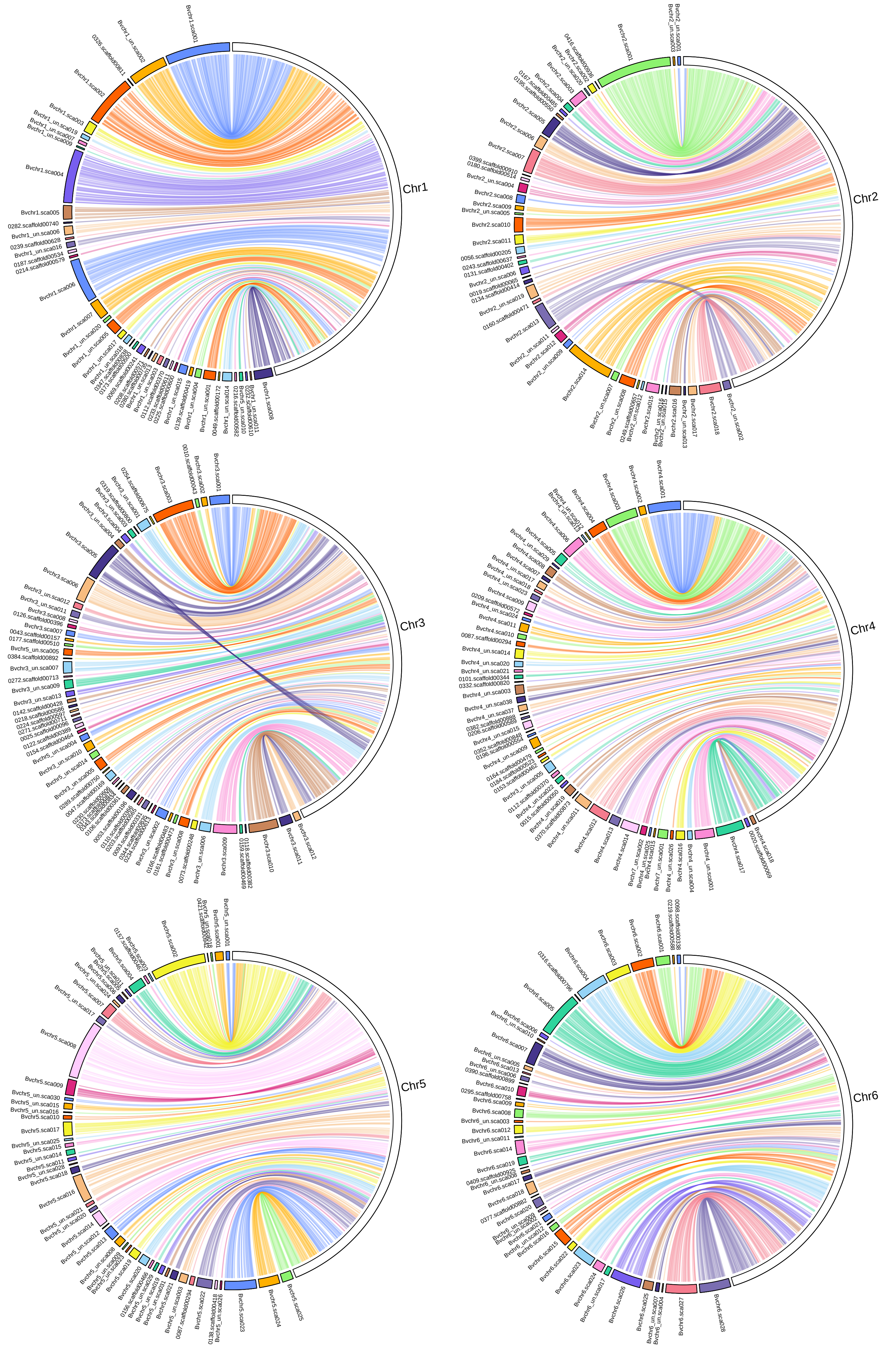
Chr1
Chr2
Chr3
Chr4
Chr5
Chr6

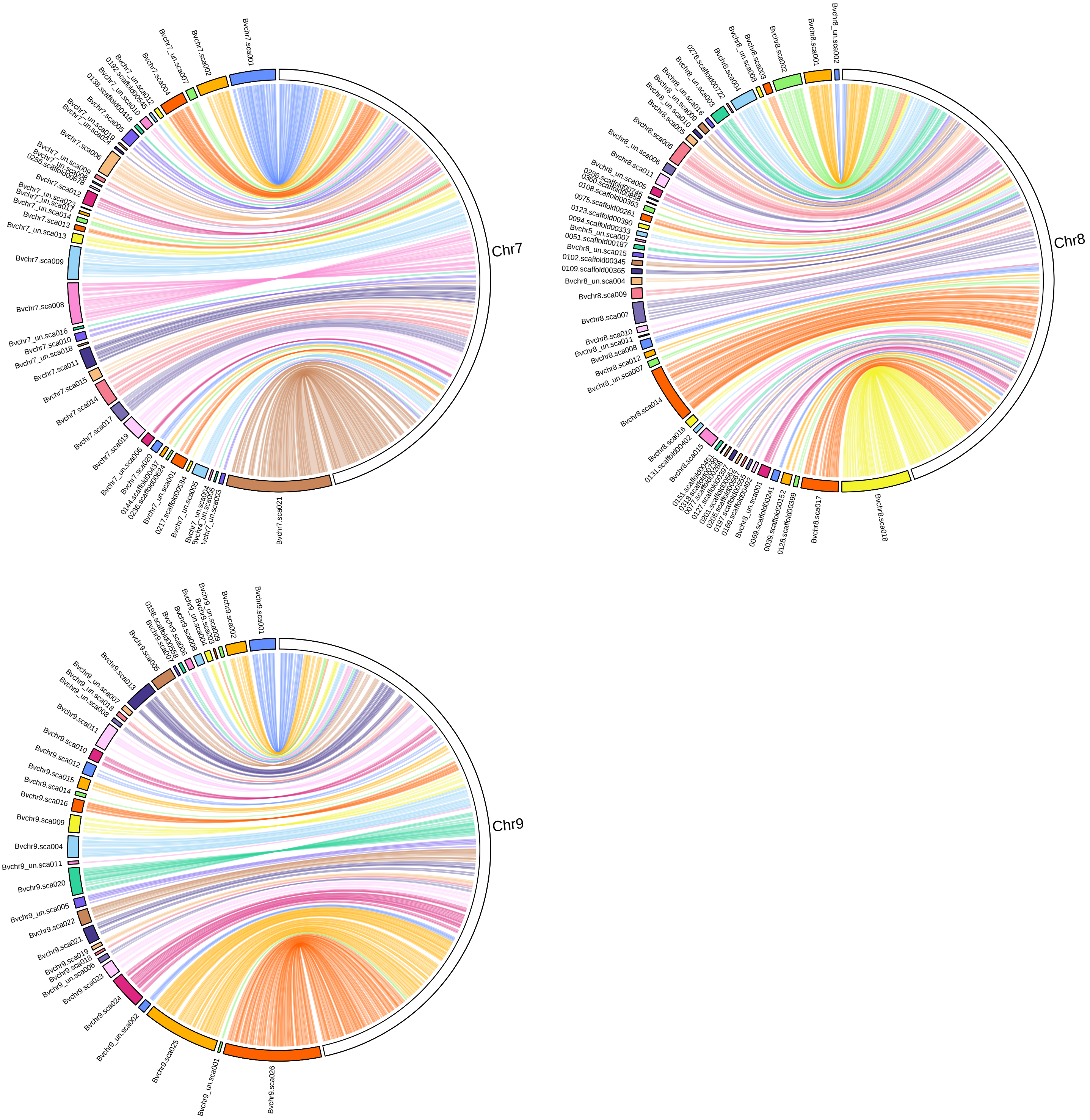

Suppl. Figure 1: Comparison of RefBeet-1.2 scaffolds (left side, coloured) and pseudochromosomes 1-9 of RefBeet-3.0 (right side, white).

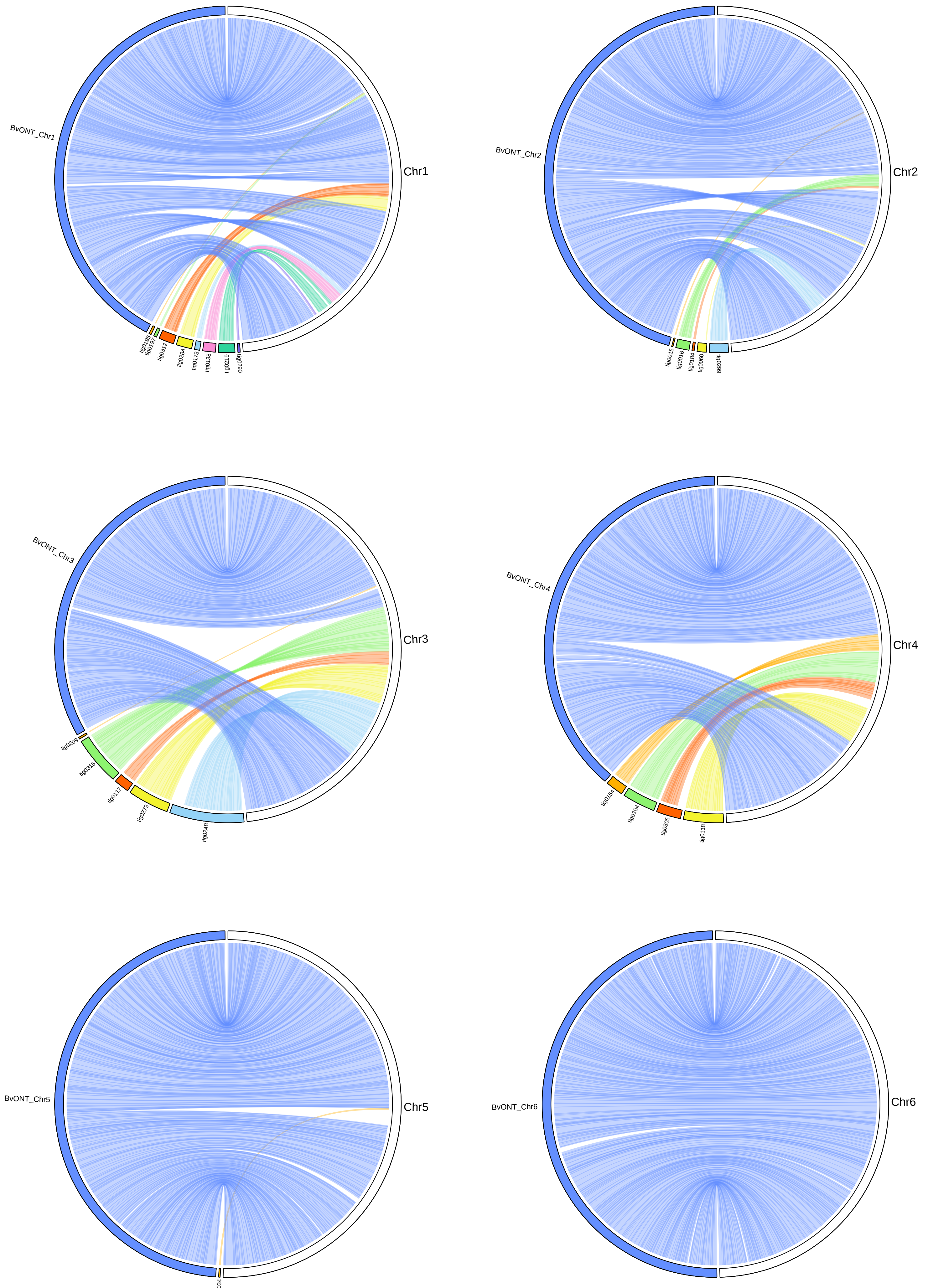
BvONT_Chr1
Chr1
tig0195
tig0197
tig0312
tig0284
tig0173
tig0138
tig0219
tig0290
BvONT_Chr2
Chr2
tig0015
tig0016
tig0184
tig0060
tig0299
BvONT_Chr3
Chr3
tig0209
tig0315
tig0117
tig0273
tig0248
BvONT_Chr4
Chr4
tig0154
tig0304
tig0305
tig0118
BvONT_Chr5
Chr5
tig0034
BvONT_Chr6
Chr6

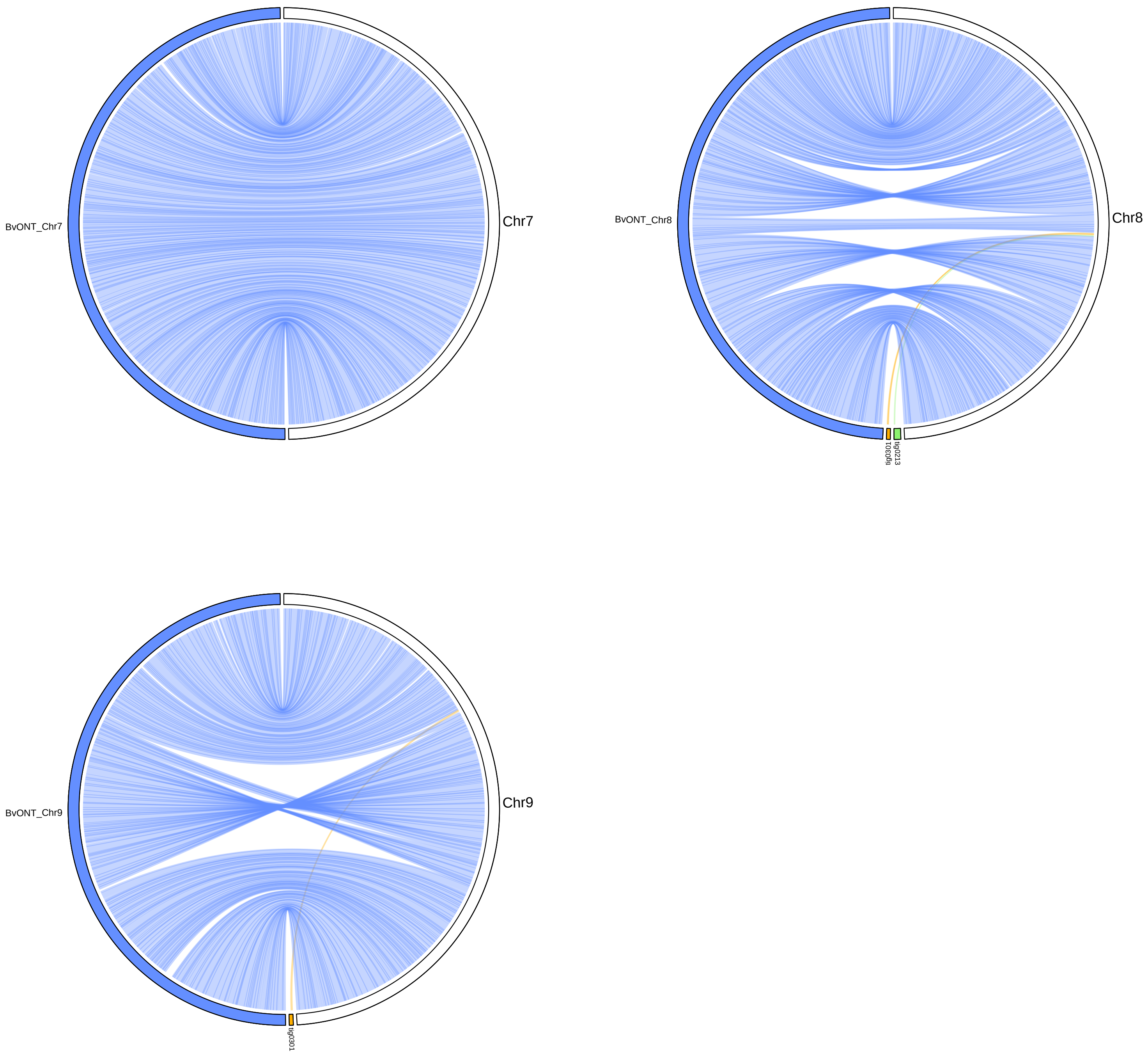

Suppl. Figure 2: Comparisons of 2320BvONT (left side, coloured) and pseudochromosomes 1-9 of RefBeet-3.0 (right side, white).

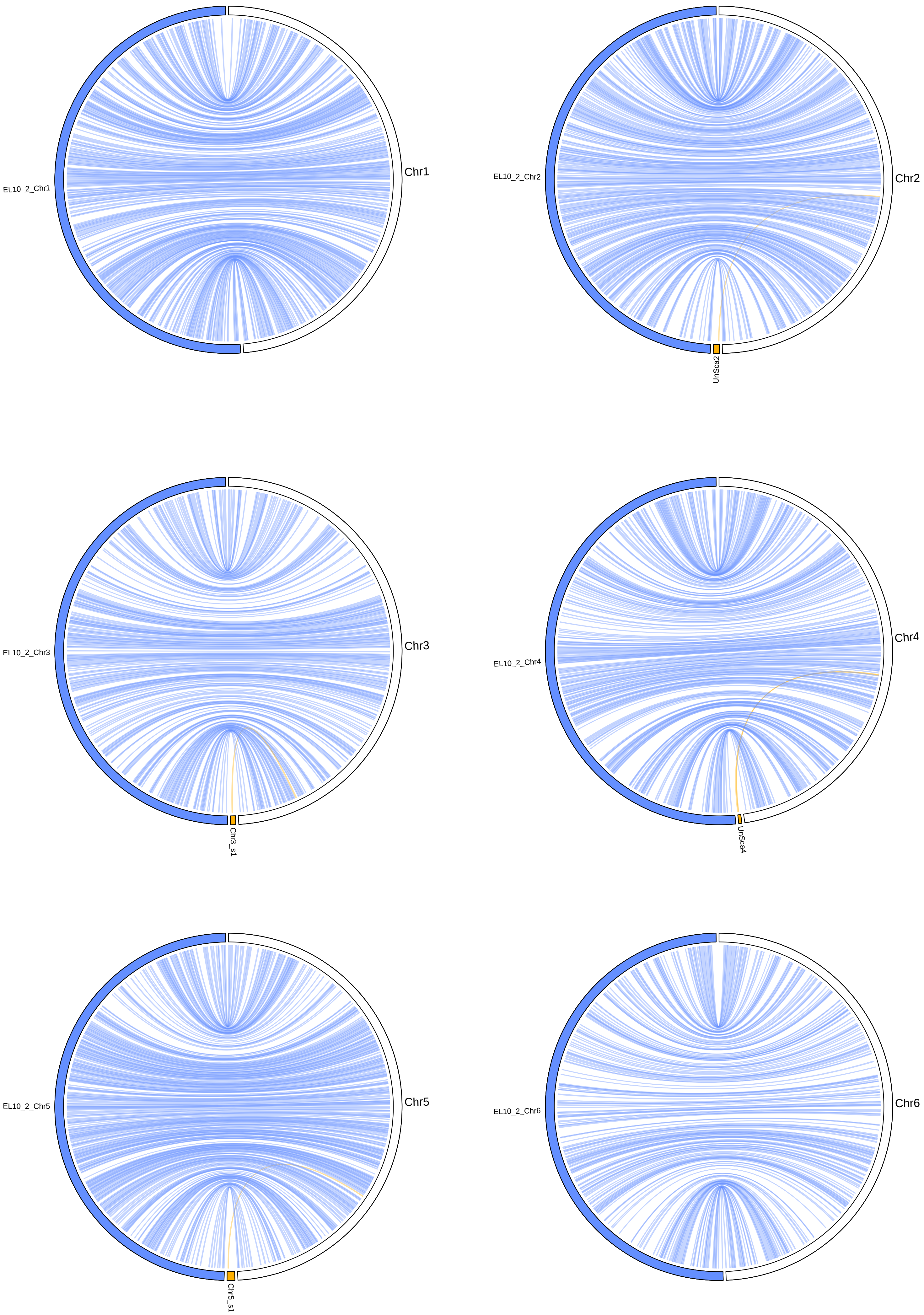
EL10_2_Chr1
Chr1
EL10_2_Chr2
Chr2
UnSca2
EL10_2_Chr3
Chr3
Chr3_s1
EL10_2_Chr4
Chr4
UnSca4
EL10_2_Chr5
Chr5
Chr5_s1
EL10_2_Chr6
Chr6

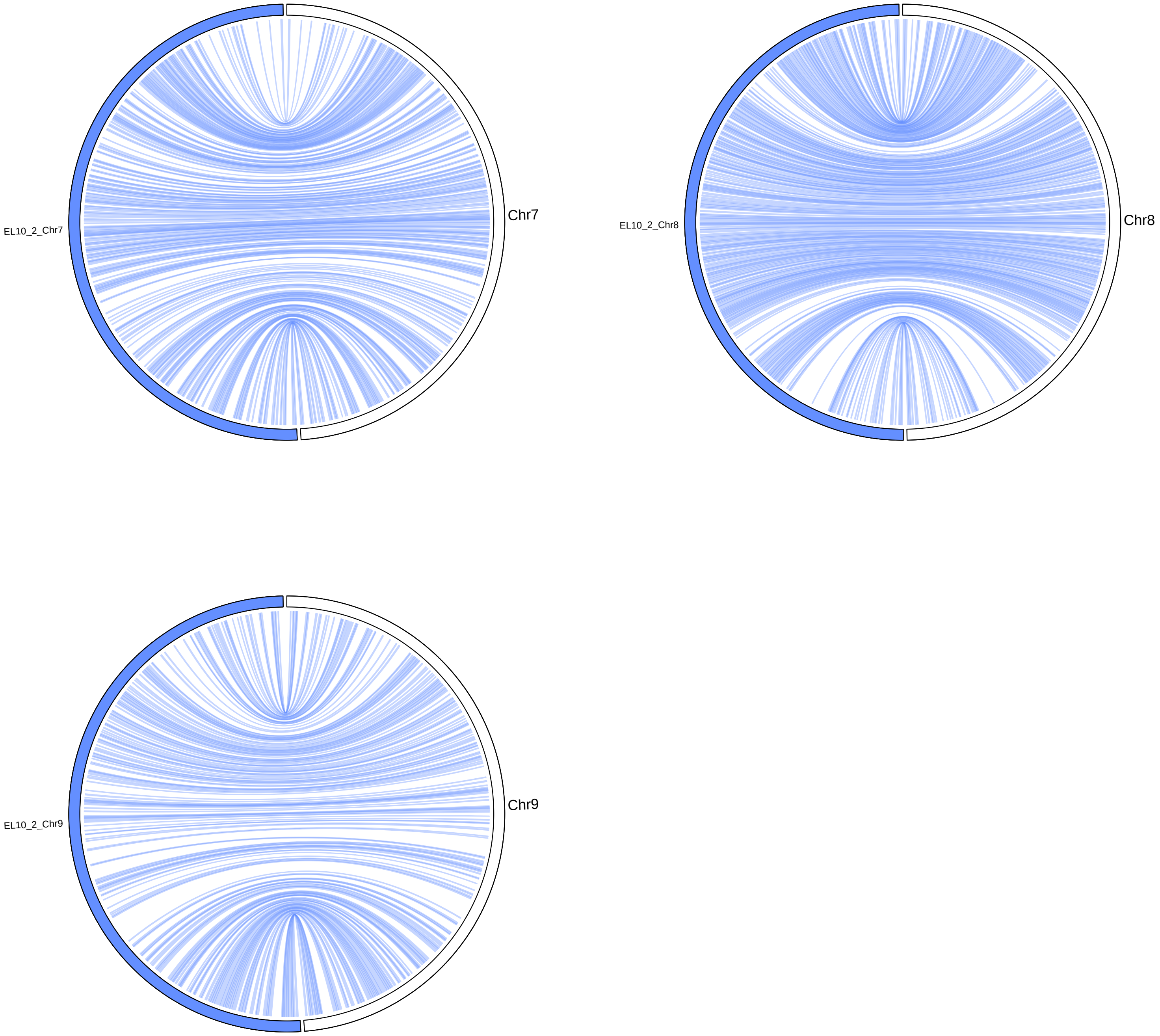

Suppl. Figure 3: Comparisons of EL10.2 (left side, coloured) and pseudochromosomes 1-9 of RefBeet-3.0 (right side, white).

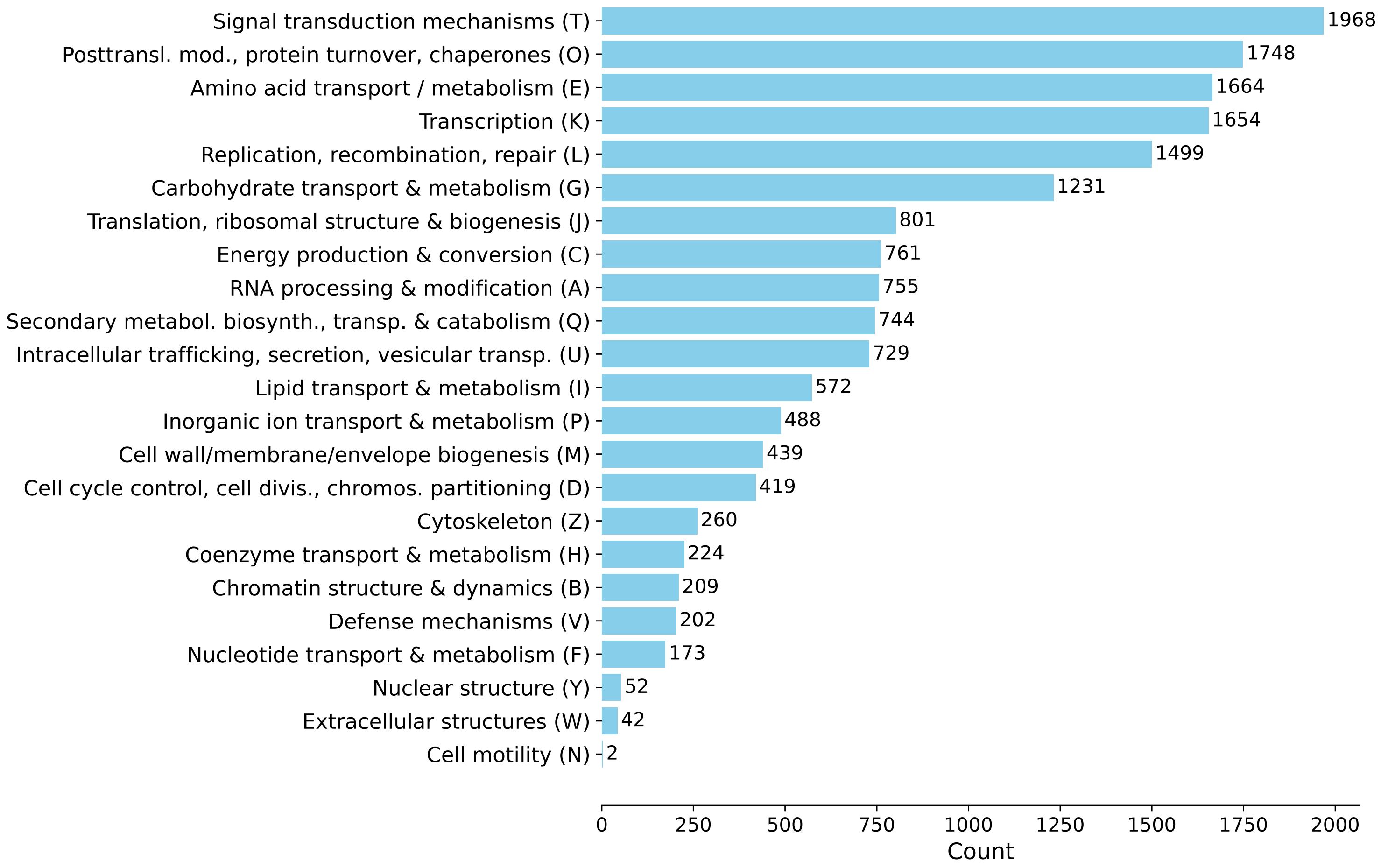

Suppl. Figure 4: Most frequent COGs' (Clusters of Othologous Genes) functional categories (omitting the category "Function unknown") assigned to BeetSet-3 genes as obtained from the eggNOG pipeline.

file name: RefBeet-3.0.fa
sequences: 3105
total length: 647616799 bp (645984020 bp excl N/X-runs)
GC level: 35.72 %
bases masked: 447421331 bp (69.09 %)

	number of elements*	length occupied	percentage of sequence
Retroelements	247708	186542040 bp	28.80 %
SINEs:	0	0 bp	0.00 %
Penelope:	0	0 bp	0.00 %
LINEs:	27013	18488089 bp	2.85 %
CRE/SLACS	0	0 bp	0.00 %
L2/CR1/Rex	0	0 bp	0.00 %
R1/LOA/Jockey	0	0 bp	0.00 %
R2/R4/NeSL	0	0 bp	0.00 %
RTE/Bov-B	1513	550414 bp	0.08 %
L1/CIN4	25500	17937675 bp	2.77 %
LTR elements:	220695	168053951 bp	25.95 %
BEL/Pao	0	0 bp	0.00 %
Ty1/Copia	81229	68783107 bp	10.62 %
Gypsy/DIRS1	52802	63861035 bp	9.86 %
Retroviral	0	0 bp	0.00 %
DNA transposons	24632	15541993 bp	2.40 %
hobo-Activator	3598	1638420 bp	0.25 %
Tc1-IS630-Pogo	1152	508754 bp	0.08 %
En-Spm	0	0 bp	0.00 %
MULE-MuDR	7028	5012292 bp	0.77 %
PiggyBac	0	0 bp	0.00 %
Tourist/Harbinger	6711	2231528 bp	0.34 %
Other (Mirage, P-element, Transib)	0	0 bp	0.00 %
Rolling-circles	7995	2760559 bp	0.43 %
Unclassified:	577613	234050949 bp	36.14 %
Total interspersed repeats:		436134982 bp	67.34 %
Small RNA:	0	0 bp	0.00 %
Satellites:	0	0 bp	0.00 %
Simple repeats:	110563	7667890 bp	1.18 %
Low complexity:	16080	857900 bp	0.13 %

* most repeats fragmented by insertions or deletions have been counted as one element

Suppl. Table 3: Repeat analysis of RefBeet-3.0.
